# Physical Performance and Quality of Life in Older Adults: Is There Any Association between Them and Potential Drug Interactions in Polymedicated Octogenarians?

**DOI:** 10.3390/ijerph16214190

**Published:** 2019-10-29

**Authors:** Zoraida Verde, Laura García de Diego, Luis M. Chicharro, Fernando Bandrés, Verónica Velasco, Teresa Mingo, Ana Fernández-Araque

**Affiliations:** 1 Department of Biochemistry, Molecular Biology and Physiology, Universidad de Valladolid, Campus Duques de Soria, 42004 Soria, Spain; 2 Department of Nursery, Universidad de Valladolid, Campus Duques de Soria, 42004 Soria, Spain; inia.laura@gmail.com (L.G.d.D.); afa@enf.uva.es (A.F.-A.); 3 Cátedra Complutense Diagnostic and Innovation, Universidad Complutense, 28040 Madrid, Spain; luismi.chicharro@gmail.com (L.M.C.); fbandres@med.ucm.es (F.B.); 4 Department of Toxicology and Health Sanitary, Universidad Complutense, 28040 Madrid, Spain; 5 Department of Nursery, Universidad de Valladolid, 47005 Valladolid, Spain; veronica.velasco.gonzalez@uva.es; 6 Department of Surgery, Ophthalmology, Otorhinolaryngology and Physiotherapy, Universidad de Valladolid, Campus Duques de Soria, 42004 Soria, Spain; tmingo@cir.uva.es

**Keywords:** cytochromes, drug interactions, octogenarians, polypharmacy, mobility, hand grip strength, ISEK test, quality of life

## Abstract

Older adults are at increased risk of several cytochrome P450 (CYP) drug interactions that can result in drug toxicity, reduced pharmacological effect, and adverse drug reactions. This study aimed to assess the prevalence of potential CYP interactions referring to the most clinically relevant drugs and exploring the relationship between them and quality of life and physical performance in Spanish octogenarians. Institutionalized and community-dwelling octogenarians (*n* = 102) treated at three primary care centers, were recruited by a research nurse. Anthropometric measurements, chronic diseases, prescribed drugs, quality of life, physical performance, mobility skills, hand grip strength and cognitive status data were collected. Potential CYP drug-drug interactions (DDIs) were selected referring to the main CYP implicated in their metabolism. The 72.2% of recruited octogenarians presented potentially inappropriate CYP inhibitor-substrate or CYP inductor-substrate combinations. Analyzing the EuroQol Visual Analogue scale (EQ-VAS) results, patients with a potential CYP DDI perceived worse health status than patients without it (*p* = 0.004). In addition, patients with a potential CYP DDI presented worse exercise capacity, kinesthetic abilities, or mobility than those who didn’t present a potential interaction (*p* = 0.01, *p* = 0.047, and *p* = 0.02, respectively). To investigate and control factors associated with loss of muscle strength and poor quality of life, polypharmacy and DDIs could help institutions in the management of physical frailty.

## Key Points:

The 72.2% of recruited octogenarians presented potentially inappropriate cytochrome P450 (CYP) inhibitor-substrate or CYP inductor-substrate combinations.

Patients with a potential CYP drug-drug interaction (DDI) perceived worse health status.

Patients with a potential CYP DDI presented worse exercise capacity, kinesthetic abilities, or mobility.

## 1. Introduction

Polypharmacy is a term used to describe the situation in which a patient is taking five or more drugs [1]. It often occurs among elderly people and is associated with an increased risk of drug-drug interactions (DDIs), which impact on patient health, effectiveness of drugs, and increased medical costs [2].

Drug metabolism via the cytochrome P450 (CYP) system has emerged as an important determinant in the incidence of several DDIs that can result in drug toxicities, reduced pharmacological effect, and adverse drug reactions [2,3,4].

The CYP enzymes are a superfamily of mono-oxigenases that metabolize many different drugs. Approximately 75% of drugs are cleared by phase I hepatic metabolism and about 70% of these involve metabolism by CYP enzymes [5]. Potentially serious adverse effects may occur when high plasma levels of a substrate drug result from the co-administration of a drug that inhibits its metabolism.

Based on this background, to identify if the involved drugs act as enzyme substrates, inducers, or inhibitors can prevent the occurrence of clinically significant interactions. Other factors such as co-morbidity, age, gender, genetics, polypharmacy, or prescribed drugs with narrow therapeutic index are related to higher DDIs probability [6,7].

Elderly people are at increased risk of adverse events due to CYP DDIs, renal clearance is reduced and despite of hepatic enzyme activity loss, changes in oxygen diffusion of hepatic endothelial are the main problem [8]. The second factor is that patients in this age group are frequently on long-term multiple medications.

Given this level of complexity, the traditional clinical assessment might not provide enough information on the risk of adverse outcomes, might be necessary for a more global evaluation examining domains commonly impaired in advanced age, including functional status, to improve quality of care, and lead to a better financial resources allocation [9]. Physical performance measures have been proven to be simple and reliable tools to assess performance abilities along the full spectrum of functioning and they were associated with a number of health outcomes. In particular, among older adults, the walking speed and the hand grip strength tests were shown to correlate with mortality, hospitalizations, institutionalization, and impaired cognition and they may represent valid and reliable outcome measures for intervention studies [10].

A wide range of studies have reported drug interactions in different populations. However, most studies have not examined potential DDIs, analyzing the prevalence of clinically relevant CYP enzyme interactions and physical performance or quality of life in elderly people [11]. We decided to evaluate prevalence, types, and severity of potential CYP DDIs in octogenarians analyzing the number of CYPs implicated in drug metabolism (substrate, inhibitor, and inducer), in each patient.

In addition, polypharmacy predisposes people to functional decline, specific drugs such as statins have been linked to falls in elderly people, however the effect of polypharmacy in mobility or related skills are not well established [3,12].

The importance of potential CYP DDIs and functional impairments such as an investigation clearly lies in considering that Soria is one of the Spanish regions with increased elderly index.

The purpose of this study was to explore the relationship between potential CYP interactions and physical quality of life in octogenarians who are free of cognitive or mobility impairments in order to identify mechanisms involved in the maintenance of physical function and prevention of functional impairment among this population.

## 2. Methods

This study is a part of a cross-sectional study carried out between October and December 2016, in a sample of institutionalized and community-dwelling octogenarians.

Eligible patients for inclusion in the study were aged 80 years old or over, without a diagnosis of dementia or mobility impairments. Mini mental status exam (MMSE) was used to screen for possible cognitive issues and mobility was evaluated relating to their ability to walk without any aid for more than 2 min [13,14]. In addition, patient’s clinical stability was an inclusion criteria condition (no hospitalization within the last six months).

The study was conducted according to the guidelines laid down in the Declaration of Helsinki 2008 and approved by the Area de Salud de Burgos y Soria Ethics Committee. Retrospective use of the patient database in primary attention was approved by the institutional ethics committee. All patients signed written informed consent.

Selected participants who were being treated at three primary care centers of the Healthcare Area of Soria were interviewed by a research nurse collecting the following data: Demographics; quality of life and physical performance measures; drugs and interactions, or clinical group risk (CGR) category. CRGs category is a claims-based classification system for risk adjustment that assigns each individual to a single mutually exclusive risk group based on historical clinical and demographic characteristics to predict future use of healthcare resources.

### 2.1. Quality of Life Measures

Quality of life measures included Barthel index (BI), EuroQol 5-Dimensions (EQ-5D), and EuroQol Visual Analogue scale (EQ-VAS).

BI is used to measure performance in activities of daily living, the BI uses ten variables describing activities of daily living and mobility, a higher number is associated with a greater likelihood of being able to live at home with a degree of independence. The EQ-5D is a standardized measure of health-related quality of life (QoL) that can be used in a wide range of health conditions and treatments. The descriptive system comprises five dimensions: Mobility, self-care, usual activities, pain/discomfort, and anxiety/depression.

EQ-VAS is a quantitative measure of health as judged by the individual, the EQ-VAS records the respondent’s self-rated health on a 20 cm vertical, visual analogue scale with endpoints labelled ‘the best health you can imagine’ and ‘the worst health you can imagine [15,16].

### 2.2. Physical Performance Measures

We evaluated a fitness test battery especially for older adults: The Senior Fitness Test [17]. Physical performance measures included muscular strength, mobility skills (inter-age score to evaluate kinesthetic abilities test (ISEK test)), exercise capacity, the 2-min step test (TMS test), functional evaluation (30-s chair stand test (30-s CS test)), flexibility (sit and reach test (SR test) and back scratch), and agility and dynamic balance (foot up-and-go test)).

Muscular strength was assessed with the hand grip strength test [18]. After adjustment for hand size, three measures were performed with the dominant hand and were averaged for the analysis.

The ISEK test was used to evaluate mobility skills [19]. ISEK test combines various elements used in other mobility tests determining the frailty during aging.

The TMS test is one of many alternatives for measuring exercise capacity. It requires the patient to march in place for 2 min, lifting their knees to a marked target set on the wall set at the midpoint between the kneecap and crest of the iliac [17].

The 30-s CS test is one of the most important functional evaluation clinical tests because it measures lower body strength and relates it to the most demanding daily life activities. It consists of standing up and sitting down from a chair as many times as possible within 30 s. A standard chair (with a seat height of 40 cm) without a backrest but with armrests was used.

The SR test is used to measure hamstring and low back flexibility. It was performed using the procedures outlined in the ACSM manual [17]. Back scratch was used to assess upper body (shoulder) flexibility, which is important in tasks such as combing one’s hair, putting on overhead garments, and reaching for a seat belt.

The foot up-and-go test, developed by Rikli and Jones, measures power, speed, agility, and dynamic balance. The test involves getting out of a chair, walking 8 feet to and around a cone, and returning to the chair in the shortest time possible [20].

### 2.3. Drugs and Interactions Analyzed

We only analyzed the most frequent regular daily drugs and also implicated in the most common potentially serious drug interactions. Drugs were classified according to the international anatomical therapeutic chemical classification system (ATC). Polypharmacy was considered the used of five or more clinically indicated medications. The drug interactions were selected attending the main CYP implicated in their metabolism (CYP2B6, CYP2D6, CYP2C9, CYP2C19, CYP3A4, and CYP3A5). We classified each drug by CYPs involved in each process (substrates, inhibitors, and inducers). To evaluate the magnitude of potential DDIs in each patient, we also presented a score to quantify the susceptibilities of different enzymes to inhibition/induction by diverse substrates of the total of regular daily drugs.

The CYPs involved in pharmacokinetics of each drug were identified by the online DrugBank or Clinical Pharmacogenetics implementation Consortium and we also checked different publications in pharmacology [21,22].

### 2.4. Statistical Analysis

Demographic and clinical data were described as mean (SD) for continuous variables and frequencies (percentages) for categorical data. Kolmogorov Smirnov normality test was performed to test the normality of continuous variables and to subsequently select the statistical tests.

Independent sample t-tests were used for continuous variables and chi-square tests for categorical variables. Associations between physical performance measures (independent variables) with dependent variables were analyzed with binary logistic regression. Data were analyzed with the PASW/SPSS Statistics 20.0 (SPSS Inc, Chicago, IL, USA) program. Statistical significance was set at *p* < 0.05.

### 2.5. Ethics Approval and Consent to Participate

The study was approved by the ethics committee “Comité de Ética de la Investigación con medicamentos de Área de Salud de Burgos y Soria”, committee’s reference number (Ref. CEIC 1446). Written consent was obtained from all participants.

## 3. Results

In this study, 102 octogenarians (44.1% males) were enrolled; 41.1% were institutionalized and 58.9% were community-dwelling. The mean of chronically consumed drugs by patients was 6.2 (SD = 3.44) and prevalence of polypharmacy was 67.6%. We did not observe any relationship between gender or age and number of prescribed drugs (*p* = 0.938 and *p* = 0.347, respectively). Institutionalized octogenarians were older (88.9 (SD = 3.28); 87.1 (SD = 4.17), *p* = 0.03, respectively), and used on average significantly more drugs than community-dwelling people (7.4 (SD = 3.33); 5.3 (SD = 3.26), *p* = 0.003, respectively). Attending BI, the percentage of participants with low dependent/independent performance in activities of daily living was of 73.3%. For CGR classification, 33.3% of participants presented pluripathologic situation or chronic diseases, however we did not find any association between CGR classification and age, gender, consumed drugs, quality of life, or physical performance (data not shown). On the other hand, we found an association between CRG and BI (*p* < 0.001) and it was predictable.

### 3.1. Potential CYP Interactions

Thirty-three drugs were assessed attending prevalence and DDIs, ten drugs presented high CYP450 inhibitory promiscuity (i.e., inhibition of multiple enzymes by a single compound) and twenty-three presented low CYP450 inhibitory promiscuity (Table 1).

Attending prescribed drugs, the most common CYP substrates involved CYP3A4 (77.7% of patients) followed by CYP2C19 (48.8%), CYP2C9 (44.4%), CYP2D6 (10.0%), CYP3A5 (5.6%), and CYP2B6 (1.1%).

In our study, we found a high frequency of potential DDIs. Potentially inappropriate CYP inhibitor-substrate or CYP inductor-substrate combinations were found in 72.7% of participants.

The most common CYP substrate-inhibitor combinations involved CYP3A4 (64.7% of patients), followed by CYP2C19 (48.0%), CYP2C9 (33.2%), and CYP2D6 (4.9%). In the case of CYP3A5 and CYP2B6, we did not find any potential inhibition interaction in studied patients.

### 3.2. Quality of Life and Physical Performance and Potential CYP DDIs

Categorizing patients in two groups, without and with a potential CYP DDI, the most significant differences were observed in the total number of prescribed drugs (*p* < 0.001), percentage of polypharmacy (*p* < 0.001) and percentage of high CYP inhibitory promiscuity medications (*p* < 0.001) (Table 2).

Performance in activities of daily living was similar between two groups (*p* = 0.444). Analyzing the EQ-VAS results, patients with a potential CYP DDI perceived worse health status than patients without it (*p* = 0.004) with a low effect size η^2^ (0.094).

Out of all physical performance tests, the TMS test for measuring exercise capacity showed the strongest association with a potential CYP DDI (*p* = 0.011; 4.52 (1.41–14.45)). We observed differences in kinesthetic abilities between two groups, patients with a potential CYP interaction spent more time, 141.1 s, in the ISEK test (*p* = 0.047) with a low effect size η^2^ (0.052). In addition, mobility skills measured by foot up and go test showed an association with a potential CYP DDI (*p* = 0.021; 3.58 (1.21–10.61)).

The other physical performance measures did not show any association with a potential CYP DDI. There were no differences in age or gender for patients without or with potential CYP DDI (see Table 2).

## 4. Discussion

Polypharmacy is a phenomenon acquiring extreme dimensions, particularly in elderly people, also in our state. Predicting CYP DDIs in a particular patient and the consequent response are mandatory, however it is still a vision and far away from application in routine clinical practice in Spain [23].

This study shows high prevalence of potential DDIs in octogenarian population, specifically in relation to clinically relevant CYP drug combinations. CYP3A4 is the most important drug metabolizing enzyme in adults, between its substrates are included a big number of therapeutic drugs as well as many endogenous substances. Potentially inappropriate CYP3A4 inhibitor-substrate combinations were found in 64.7% of patients. This combination can result in increased plasma levels of CYP3A4 substrates such as simvastatin or atorvastatin, and statins are associated with myopathy and possibly impaired muscle function [24]. For CYP2C19 and CYP2C9 potentially inappropriate CYP inhibitor-substrate combinations were found in 48.0% and 33.2% of patients, respectively. CYP2C19, CYP2C9, and CYP2D6 DDIs have been related to falls as a frequent type of adverse drug reactions causing significant morbidity and mortality in the elderly [25].

Keeping in mind drugs with a narrow therapeutic index as acenocoumarol, the aforementioned drug was prescribed in 19.3% of patients and 70.5% of them presented intake of potential inhibitors of CYP2C9. In addition, omeoprazol was prescribed in 19.3% of patients. Proton-pump inhibitors (PPIs) are a widely prescribed class of medications used to treat acid-related disorders and use has significantly increased over the last few decades. PPIs are often inappropriately prescribed and several studies have been published demonstrating associations between longer duration of PPI therapy and a number of adverse effects that are a concern in older adults [26].

Hence, we observed that octogenarians taking potentially inappropriate CYP inhibitor-substrate pairs had a lower quality of life or physical performance and were taking significantly more prescribed drugs. This is in agreement with previous findings that the risk of CYP mediated DDIs increases with the number of drugs [11,27].

The EQ-VAS showed notably low scores for the patients’ own assessment of health-related quality of life in relation to polypharmacy or potential CYP interactions. The average score on the EQ-VAS (63.12 ± 17.37) in our sample is consistent with findings from other national studies [28]. Montiel Luque et al. also reported that patients who take a larger number of drugs presented the worst results for the EQ-VAS, however they did not analyze potential CYP DDIs. In our study, the EQ-VAS was the factor that showed the strongest relationship with potential CYP DDIs.

On the other hand, according to our results, poorer mobility skills, exercise capacity and agility and dynamic balance are associated with potential CYP DDIs among octogenarians. To our knowledge, this is the first study analyzing a complete background of quality of life and physical performance tests and potential CYP DDIs in octogenarians. Physical performance measures have proven to be valid and reliable and they are now widely used in geriatric research because of their sensitivity to change over time and predictive validity for important health outcomes such as self-perceived health, hospitalization, falls, mortality, and onset of disability in several populations [29].

Previously, other authors have demonstrated that physical activity or muscular strength were inversely associated to polypharmacy in elderly patients [30,31,32]. Our results are also in agreement to aforementioned authors, grip strength test, and ISEK test were significantly lower in patients with polypharmacy (*p* = 0.028 and *p* = 0.010, respectively) (Data not shown). In the case of ISEK test, it determines frailty during aging and adds a precise movement of the hand and is a dynamic test complementary to grip strength test, there is no previous studies that included it.

On the other hand, for TMS test, 30-s CS test, SR test, back scratch test, and foot up-and-go test, we have not found any study including them.

Therefore, only a few studies have addressed the association between physical performance measures and polypharmacy in the elderly. However, there are no data about potential DDIs and functional fitness.

Assessing potential DDIs, the functional fitness performance of older adults, the physiologic capacity to perform normal everyday activities safely and independently is mandatory in order to identify populations at-risk.

We have also observed a relationship between a higher number of prescribed drugs, higher potential CYP interactions, and worse quality of life. In the case of elderly patients, it is usual to treat a previous health effect caused by a prescribed drug with a new medication, so potential CYP interactions are a serious problem.

The fact that in elderly population, the ability to clear drugs is significantly decreased makes the situation worse. In addition to the inhibition of enzymes due to the concomitant intake of several potentially interacting drugs, polymorphisms of drug metabolizing CYPs with functional and clinical correlates must be considered within its pharmacological context [33].

We know that our study has several limitations, the number of patients is low, this is why we have considered it is a pilot study to be validated. On the other hand, our sample is homogeneous, including institutionalized and community-dwelling people. Another limitation is that we didn’t recover the dose of medications used by the patient. However, we think that our findings have important implications for elderly patients who suffer from polypharmacy. From a public health point of view, to reduce the polypharmacy is essential [34].

Knowledge of potential DDIs provides clinically useful information for optimizing polypharmacy in elderly patients. Potential adverse effects resulting from interactions between CYP inhibitors or inducers and substrates are predictable. Many factors including, genetic biomarkers and drug-drug interactions also might help identifying patients at high risk for drug-induced adverse effects. In addition, further studies analyzing CYPs implicated and the impact of genomic variation on the medication-related interactions in disability in elderly people are needed. To investigate and control factors associated with loss of muscle strength and poor quality of life could help institutions in the management of physical frailty.

## 5. Conclusions

A number of significant CYP DDIs and lower physical performance and quality of life were found to be associated with polypharmacy among octogenarians who were taking chronic medications. The present study adds to the growing evidence that a significant proportion of older adults are exposed to multiple medication related issues. Physical performance and quality of life measurements in addition to morbidity should be included in health systems as a measure of health outcome in this age group. More stringent guidelines are needed. In addition, healthcare providers should be vigilant of such usage to prevent any interactions with prescribed medications.

## Figures and Tables

**Table 1:** Medications most implicated in drug-drug interactions and prevalence.

Medication	ATC Code	Prevalence (%)	CYP †	CYP Inhibitory Promiscuity
Omeoprazol	A02BC01	45.4	CYP2C19	High
Acetaminophen	N02BE01	38.6	CYP3A4	Low
Acenocumarol	B01AA07	19.3	CYP2C9	Low
Simvastatin	C10BX04	19.3	CYP3A4	Low
Lorazepam	N05BA06	18.2	CYP3A4	Low
Acetylsalicylic Acid	N02BA51	17.0	CYP2C9	Low
Torasemide	C03CA04	17.0	CYP2C9	Low
Furosemide	C03CA01	15.9	-	Low
Enalapril	C09AA02	14.8	CYP3A4	Low
Insuline	A10AB01	14.8	-	Low
Tamsulosin	G04CA02	13.6	CYP3A4/2D6	Low
Allopurinol	M04AA01	12.5	-	High
Folic Acid	B03AE01	10.2	-	Low
Atorvastatin	C10AA05	9.1	CYP3A4	High
Bisoprolol	C07BB07	9.1	CYP3A4	Low
Chlolecalciferol	M05BB08	9.1	CYP3A4	Low
Colchicine	M04AC01	9.1	CYP3A4	Low
Metformin	A10BD18	9.1	-	Low
Spironolactone	C03DA01	9.1	-	Low
Amlodipine	C09DX01	7.9	CYP3A4	High
Clopidogrel	B01AC04	6.8	CYP3A4/2C19	High
Digoxin	C01AA05	6.8	CYP3A4	Low
Pentoxifylline	C04AD03	6.8	CYP3A4	Low
Tramadol	N02AX52	6.8	CYP3A4/2D6	High
Valsartan	C09CA03	6.8	CYP2C9	High
Hydroxyzine	N05BB51	5.7	-	Low
Sertraline	N06AB06	5.7	CYP3A4/2C19	High
Metamizole	N02BB72	4.5	-	Low
Zolpidem	N05CF02	4.5	CYP3A4	Low
Apixaban	B01AF02	3.4	CYP3A4/5	High
Enoxaparin	B01AB05	3.4	-	Low
Amiodarone	C01BD01	2.3	CYP2C8	High

Note: ATC: Anatomical Therapeutic Chemical. CYP: Cytochrome P450. † Substrate: Major CYP involved.

**Table 2:** Characteristics of the study population.

Characteristics	Without CYP Interaction *n* = 29	With CYP Interaction *n* = 73	Adjusted *p*-Value
Age, mean (SD)	87.8 (4.0)	87.8 (3.9)	0.108
Male (%)	54.2	45.3	0.308
CRG (% pluripathologic or chronic diseases)	25.0	31.3	0.384
Institutionalized (%)	25.0	45.3	0.066
Total drugs used, mean (SD)	2.8 (2.1)	7.4 (3.0)	**<0.001**
Polypharmacy (%)	29.2	76.6	**<0.001**
CYP inhibitory promiscuity (%)	8.3	76.6	**<0.001**
Quality of Life			
Barthel index (%) ^a^	79.2	74.6	0.444
EQ-5D, mean (SD)	0.717 (0.18)	0.602 (0.25)	0.052
EQ-VAS, mean (SD)	72.8 (12.8)	60.5 (18.2)	**0.004**
Physical Performance			
Hand grip, mean (SD)	20.7 (8.5)	17.0 (7.8)	0.066
ISEK test, mean (SD)	117.9 (30.0)	141.1 (50.3)	0.047
TMS test (%) ^b^	35.3	71.2	0.010
30-s CS test (%) ^c^	31.8	55.2	0.052
SR test (%) ^d^	27.3	34.5	0.369
Back scratch test (%) ^e^	86.4	94.8	0.204
Foot up and go test (%) ^f^	50.0	78.2	0.020

Note: Values are percentages for categorical data or mean and standard deviation for continuous data. SD: Standard deviation; CRG: Clinical Risk Groups; CYP: Cytochrome P450; EQ-VAS: EuroQol Visual Analogue scale; ISEK: Inter-age score to evaluate kinesthetic abilities; TMS: 2-min step; CS: Chair stand; SR: Sit and reach. ^a^ percentage of independency/low dependency. ^b^ percentage of poor/good exercise capacity. ^c^ percentage of poor/medium-high body strength. ^d^ percentage of poor/medium-high hamstring and low back flexibility. ^e^ percentage of poor/medium-high shoulder flexibility. ^f^ percentage of poor/medium-high mobility skills. Statistically significant variables are in bold.

## Data Availability

The data that support the findings of this study are available from the corresponding author on request.

## List of Abbreviations

ATC	Anatomical Therapeutic Chemical Classification System
BI	Barthel index
CRG	Clinical Risk Groups
CYP	Cytochrome P450
DDIs	Drug-drug interactions
EQ-VAS	EuroQol Visual Analogue scale
ISEK test	Inter-age score to evaluate kinesthetic abilities test
SR test	Sit and reach test
TMS test	2-min step test
30-s CS test	30-s chair stand test
